# Association of State Insurance Mandates for Fertility Treatment With Multiple Embryo Transfer After Preimplantation Genetic Testing for Aneuploidy

**DOI:** 10.1001/jamanetworkopen.2022.51739

**Published:** 2023-01-27

**Authors:** Bronwyn S. Bedrick, Katelin B. Nickel, Joan K. Riley, Tarun Jain, Emily S. Jungheim

**Affiliations:** 1 Department of Gynecology and Obstetrics, Johns Hopkins University School of Medicine, Baltimore, Maryland; 2 Division of Infectious Diseases, Department of Medicine, Washington University School of Medicine in St Louis, Missouri; 3 Division of Reproductive Endocrinology and Infertility, Department of Obstetrics and Gynecology, Northwestern University Feinberg School of Medicine, Chicago, Illinois

## Abstract

**Importance:**

Multiple gestation is one of the biggest risks after in vitro fertilization (IVF), largely due to multiple embryo transfer (MET). Single embryo transfer (SET) uptake has increased over time and has been attributed to various factors, such as mandated insurance coverage for IVF and preimplantation genetic testing for aneuploidy (PGT-A).

**Objective:**

To investigate whether mandates for IVF insurance coverage are associated with decreased use of MET after PGT-A.

**Design, Setting, and Participants:**

This cohort study was conducted using data on embryo transfers reported to the Society for Assisted Reproductive Technology between 2014 and 2016. Data were analyzed from January to October 2021.

**Exposures:**

State-mandated coverage for fertility treatment and type of cycle transfer performed (PGT-A, untested fresh, and untested frozen).

**Main Outcomes and Measures:**

Use of MET compared with SET, live birth, and live birth of multiples.

**Results:**

There were 110 843 embryo transfers (mean [SD] patient age, 34.0 [4.5] years; 5520 individuals identified as African American [5.0%], 10 035 as Asian [9.0%], 5425 as Hispanic [4.9%], 45 561 as White [41.1%], and 44 302 as other or unknown race or ethnicity [40.0%]); 17 650 transfers used embryos that underwent PGT-A. Overall, among transferred embryos that had PGT-A, there were 9712 live births (55.0%). The odds of live birth were 70% higher with MET vs SET after frozen embryo transfer with PGT-A (OR, 1.70; 95% CI, 1.61-1.78), but the risk of multiples was 5 times higher (OR, 5.33; 95% CI, 5.22-5.44). The odds of MET in cycles with PGT-A in states with insurance mandates were 24% lower than in states without mandates (OR, 0.76; 95% CI, 0.68-0.85).

**Conclusions and Relevance:**

This study found that despite the promise of using SET with PGT-A, MET after PGT-A was not uncommon. This practice was more common in states without insurance mandates and was associated with a high risk of multiples.

## Key Points

**Question:**

Is state-mandated insurance coverage for in vitro fertilization associated with decreased use of multiple embryo transfer (MET) after preimplantation genetic testing for aneuploidy (PGT-A)?

**Findings:**

In this cohort study of 110 843 embryo transfers, the odds of MET were 24% lower in cycles with PGT-A in states with fertility mandates than in states without these mandates. While the odds of live birth were 70% higher with MET vs single embryo transfer after PGT-A, the risk of multiples was 5 times higher.

**Meaning:**

This study found that MET after PGT-A was more common in states without fertility insurance mandates and was associated with increased risk of multiples.

## Teaser

This cohort study investigates the association of state-mandated insurance coverage for in vitro fertilization with rates of multiple embryo transfer after preimplanation genetic testing for aneuploidy.

## Introduction

Multiple gestation is the most common risk associated with fertility treatment.^1^ Pregnancies with multiples are at significant risk for hypertensive disorders,^2^ gestational diabetes,^3^ and maternal mortality,^4^ as well as prematurity,^5^ cerebral palsy,^6^ and neonatal and fetal demise.^6^ Furthermore, the health care cost associated with multifetal gestations is more than 5 times that associated with singleton gestations.^7^

For individuals who require in vitro fertilization (IVF), the risk of multiple gestation can be controlled by limiting the number of embryos transferred. Accordingly, the American Society for Reproductive Medicine (ASRM) and Society for Assisted Reproductive Technology (SART) publish guidelines on the suggested number of embryos to transfer in IVF.^8^ These guidelines incorporate several factors, and over time they have placed increasing emphasis on single embryo transfer (SET).^9,10,11,12^

Preimplantation genetic testing for aneuploidy (PGT-A) was pioneered as a technology to select euploid embryos for transfer^13,14^ and to promote SET use.^15^ State mandates for IVF insurance are associated with safe IVF practice patterns and outcomes, including fewer embryos transferred per cycle, increased use of SET, and decreased risk of multiple gestation after IVF.^16^ However, the association between state-mandated insurance and the number of embryos transferred after PGT-A has not been studied. We therefore sought to investigate the association between state-mandated insurance for fertility coverage and use of multiple embryo transfer (MET) after PGT-A.

## Methods

This study was approved by the SART Research Committee and the institutional review board at Washington University School of Medicine in St. Louis with a waiver of informed consent as research not involving human participants. The Strengthening the Reporting of Observational Studies in Epidemiology (STROBE) reporting guideline for cohort studies was followed.

### Data Set

Data were obtained from the 2014 to 2016 SART Clinic Outcome Reporting System (CORS) registry. In this registry, clinic data are collected through voluntary submission, verified by SART, and reported to the Centers for Disease Control and Prevention in compliance with the Fertility Clinic Success Rate and Certification Act of 1992.^17^ The data in the SART CORS are validated annually, with approximately 10% of clinics receiving on-site visits for medical record review. In 2019, discrepancy rates of 5% or lower were found in 9 of 11 data fields selected for validation.^18^

The data set included deidentified autologous embryo transfers as detailed previously.^19^ Exclusion criteria for this study were patient age younger than 18 years or older than 50 years, missing patient state of residence and clinic state, use of nonblastocyst stage transfer, and unknown PGT status. For a more apt comparison between transfers of embryos with and without PGT-A, transferred embryos that underwent PGT for reasons other than aneuploidy (eg, monogenetic disorders) were removed from analyses. We restricted analyses to each patient’s first embryo transfer.

Embryo transfers were categorized as frozen or fresh and by use of PGT-A: PGT-A frozen embryo transfer (FET), untested FET, and untested fresh transfer. States were categorized by presence of mandated insurance coverage in the fertility clinic state, as a mandate state (Arkansas, Connecticut, Hawaii, Illinois, Maryland, Massachusetts, Montana, New Jersey, Ohio, Rhode Island, and West Virginia) or nonmandate state (remaining states and the District of Columbia).^16,20,21^ There were no changes in mandate categorization during the study period.^21^ If the clinic state was missing, the state for patient residence was used. If the clinic state was missing and the patient lived in a state with no fertility centers, the transfer was excluded. The number of embryos transferred was categorized as 1, 2, or 3 or more. Among 64 331 total pregnancies, 88 pregnancies (0.1%) originated as a multiple gestation with a singleton live birth and stillbirth of the other fetuses. A stillbirth was defined as fetal demise after 20 weeks of gestation or birth of a demised fetus weighing more than 350 g if the gestational age was not known. Information about multiple gestations with the demise of a fetus prior to 20 weeks was not available. Therefore, for this analysis, a multiple gestation was defined as having more than 1 live-born infant at delivery. Race and ethnicity were included in the analysis given racial disparities in access to and disparate outcomes after fertility treatments.^22,23^ Race and ethnicity were self-reported and listed in the same question; categories included African American or Black, American Indian or Alaskan Native, Asian, Hispanic, Native Hawaiian or other Pacific Islander, and White. Groups that constituted a small percentage (<5%) of the database (American Indian or Alaskan Native and Native Hawaiian or other Pacific Islander) and individuals who identified as multiple races were included in other race to reduce the risk of bias. Additionally, those who declined to identify and those who were not asked to identify were classified as unknown.

### Statistical Analysis

Univariate comparisons were conducted using χ^2^ and Wilcoxon rank sum tests as appropriate. To evaluate differences in the number of embryos transferred by state-mandated insurance, analysis of variance was performed. Given that 22 FETs with PGT-A involved transfer of 3 or more embryos (Table 1), the number of embryos transferred was analyzed binarily as SET vs MET. To evaluate the risk of MET, logistic regression was performed, stratified by type of embryo transfer (FET with PGT-A, fresh ET, and FET) and controlling for patient age at transfer, patient race and ethnicity, number of oocytes retrieved, infertility diagnosis, prior live birth events, and presence of insurance mandate. The chance of having a live birth was modeled using logistic regression accounting for the same variables plus body mass index (BMI; calculated as weight in kilograms divided by height in meters squared). To evaluate the risk of multiple births, logistic regression was performed controlling for MET, patient age at transfer, patient race and ethnicity, prior live birth events, and infertility diagnosis. Statistical analyses were performed with SAS statistical software version 9.4 (SAS Institute). *P* values were 2-sided, and *P* < .001 was considered statistically significant. Analysis was conducted between January and October 2021.

**Table 1.** Cycle Characteristics

Characteristic	Cycles, No. (%) (N = 110 843)			*P* value
	FET with PGT-A (n = 17 650 [16.0%])	Untested FET (n = 28 046 [25.3%])	Untested fresh (n = 65 147 [58.7%])	
Age, mean (SD), y	36.8 (3.9)	33.6 (4.6)	33.4 (4.3)	<.0001
Race and ethnicity				
African American or Black	479 (2.7)	1613 (5.8)	3428 (5.3)	<.0001
Asian	2129 (12.1)	2955 (10.5)	4951 (7.6)	<.0001
Hispanic or Latino	616 (3.5)	1687 (6.0)	3122 (4.8)	<.0001
White	6589 (37.3)	11 417 (40.7)	27 555 (42.3)	<.0001
Other or unknown^a^	7837 (44.4)	10 374 (37.0)	26 091 (40.0)	<.0001
BMI, median (IQR)^b^	24 (21-27)	24 (21-28)	25 (22-29)	<.0001
Reason for ART				
Male infertility	1667 (9.4)	5461 (19.5)	15 205 (23.3)	<.0001
Endometriosis	321 (1.8)	985 (3.5)	2668 (4.1)	<.0001
Ovulation disorder	879 (5.0)	3206 (11.4)	6020 (9.2)	<.0001
Diminished ovarian reserve	2587 (14.7)	2148 (7.7)	5208 (8.0)	<.0001
Tubal factor	526 (3.0)	2030 (7.2)	5698 (8.8)	<.0001
Uterine factor	226 (1.3)	397 (1.4)	776 (1.2)	.02
PGT	36 (0.2)	16 (0.1)	7 (0.0)	<.0001
No infertility diagnosis	0	1 (<0.1)	0	.23
Unexplained	2159 (12.2)	3816 (13.6)	12 072 (18.5)	<.0001
Other	1169 (6.6)	1358 (4.8)	2111 (3.2)	<.0001
Multiple diagnoses	7761 (44.0)	7913 (28.2)	14 511 (22.3)	<.0001
No reason for ART provided	319 (1.8)	715 (2.6)	871 (1.3)	<.0001
Prior birth events, No.				
0	12 965 (73.8)	21 229 (75.7)	52 025 (79.9)	<.0001
1	3480 (19.7)	4951 (17.7)	9417 (14.5)	
≥2	1132 (6.4)	1776 (6.3)	3659 (5.6)	
Unknown	73 (0.4)	90 (0.3)	46 (0.1)	
Oocytes retrieved, median (IQR), No.	15 (10-22)	17 (11-25)	13 (9-19)	<.0001
Prior retrievals, mean (SD), No.	1.3 (0.9)	0.9 (0.6)	0.0 (0.2)	<.0001
Embryos transferred, mean (SD), No.	1.2 (0.4)	1.5 (0.6)	1.6 (0.6)	<.0001
Embryos transferred, No.				
1	14 646 (83.0)	14 636 (52.2)	29 161 (44.8)	<.0001
2	2982 (16.9)	12 851 (45.8)	33 484 (51.4)	
≥3	22 (0.1)	559 (2.0)	2502 (3.8)	
Live birth^c^	9712 (55.0)	13 765 (49.1)	30 403 (46.7)	<.0001
Live born, No.^d^				
1	8716 (89.7)	10 949 (79.5)	23 689 (77.9)	<.0001
2	973 (10.0)	2762 (20.1)	6526 (21.5)	
≥3	23 (0.2)	54 (0.4)	188 (0.6)	
Liveborn after SET, No.^e^				
1	7651 (98.8)	6518 (98.4)	13 004 (98.2)	.0001
2	92 (1.2)	104 (1.6)	232 (1.8)	
≥3	1 (<0.01)	4 (<0.01)	3 (<0.01)	

Abbreviations: ART, assisted reproductive technology; BMI, body mass index (calculated as weight in kilograms divided by height in meters squared); FET, frozen embryo transfer; PGT, preimplantation genetic testing; PGT-A, preimplantation genetic testing for aneuploidy; SET, single embryo transfer.

^a^ The other or unknown category comprises individuals who were not asked to self-identify, who declined to self-identify, who self-identified as multiple races, or who identified as a racial group that constituted a small portion of the overall database (American Indian or Alaskan Native and Native Hawaiian or other Pacific Islander).

^b^ The median was calculated excluding 20 651 individuals with unknown BMI and 41 individuals with improbable BMIs.

^c^ The denominator is the entire study cohort of first transfers per 110 843 individuals.

^d^ The denominator is 53 880 pregnancies with 1 or more liveborn infants.

^e^ The denominator is 27 609 pregnancies resulting with 1 or more liveborn infants after SET.

Among 110 843 included cycles, 209 cycles (0.19%) had missing information about previous live births. These cycles were excluded from multivariable analyses. Race and ethnicity were missing in 42 865 cycles (38.7%). A total of 20 651 cycles (18.6%) had missing BMI. We excluded 41 cycles (0.04%) with unlikely BMI values (<14 and >70) from analyses. Multiple imputations were performed for BMI using baseline characteristics included in models. Race and ethnicity was self-reported and likely not missing at random. Therefore, imputation was not performed. Instead, these cycles were categorized as other or unknown.

## Results

Between 2014 and 2016, there were 181 813 nonbanking embryo transfers. After exclusions (eFigure in Supplement 1), there were 110 843 embryo transfers (mean [SD] patient age, 34.0 [4.5] years; 5520 individuals identified as African American [5.0%], 10 035 as Asian [9.0%], 5425 as Hispanic [4.9%], 45 561 as White [41.1%], and 44 302 as other or unknown race or ethnicity [40.0% ]). Of these, 17 650 cycles (15.9%) were FET cycles with PGT-A, 28 046 cycles (25.3%) were untested FET cycles, and 65 147 cycles (58.8%) were untested fresh embryo transfers. Among transferred embryos that had PGT-A, there were 9712 live births (55.0%). Among all transfers, 76 573 transfers (69.1%) occurred in nonmandate states. Individuals undergoing FET cycles with PGT-A were older than individuals who underwent untested FET or fresh embryo transfer cycles (mean [SD] age, 36.8 [3.9] years vs 33.6 [4.6] and 33.4 [4.3] years, respectively) (Table 1). A significantly larger proportion of Asian individuals (2129 individuals 12.1%) underwent FET with PGT-A compared with individuals with other races or ethnicities, and significantly lower proportions of African American (479 individuals [2.7%]) and Hispanic or Latino (616 individuals [3.5%]) individuals underwent FET with PGT-A (P < .0001 for all comparisons) (Table 1). Diminished ovarian reserve was the most common single indication for IVF for FET with PGT-A, and male factor infertility was the most common indication for untested fresh and frozen embryo transfer.

The mean (SD) number of embryos transferred was lower for FET with PGT-A (1.2 [0.4] embryos) than untested frozen (1.6 [0.6] embryos) and fresh (1.5 [0.6] embryos) transfer (Table 1). While most FET cycles with PGT-A were SET (14 646 cycles [83.0%]), MET was involved in 3004 cycles (17.0%). The mean number of embryos transferred increased with age for untested fresh and frozen embryo transfers (eTable in Supplement 1). For FET cycles with PGT-A, the mean number of embryos transferred was fairly consistent across age categories but was lower for the oldest age category. There was a lower proportional use of SET in nonmandate states (*P* < .0001), and this disparity was significantly different by cycle type (*P* for interaction < .0001) (Figure). For states with mandated insurance 3178 of 3669 transfers with PGT-A (86.6%) were SET compared with 11 468 of 13 981 such transfers (82.0%) in nonmandate states. State mandate was associated with a greater difference between untested fresh and frozen embryo transfers in rates of SET. For example, 12 940 of 23 341 untested fresh embryo transfers in mandate states (55.4%) were SET compared with 16 221 of 41806 such transfers in nonmandate states (38.8%). Transfer of 3 or more embryos was overall rare but more common in nonmandate states.

**Figure.**
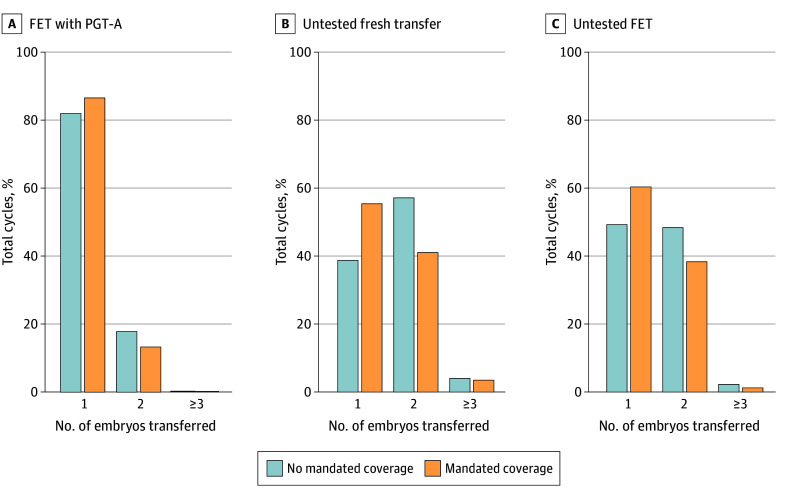
Embryos Transferred per Cycle FET indicates frozen embryo transfer; PGT-A, preimplantation genetic testing for aneuploidy.

In multivariable analysis, Hispanic or Latino individuals were significantly more likely to undergo MET than White individuals in all cycle types: 47% more likely for cycles with PGT-A (odds ratio [OR], 1.47; 95% CI, 1.19-1.82), 65% for untested FET cycles (OR, 1.65; 95%, 1.48-1.84), and 55% for untested fresh cycles (OR, 1.55; 955 CI, 1.42-1.68) (Table 2). African American individuals were 68% more likely to undergo MET than White individuals in embryo transfers with PGT-A (OR, 1.68; 95% CI, 1.33-2.11) and 78% more likely to undergo MET in untested FET cycles (OR, 1.78; 95% CI, 1.41-1.76). With increasing age, individuals were significantly less likely to undergo MET after PGT-A. The opposite trend was seen for untested frozen and fresh embryo transfers.

**Table 2.** Logistic Regression Models for Performance of MET

Characteristic	MET, OR (95% CI)		
	FET with PGT-A (n = 17 577)	Untested FET (n = 27 956)	Untested fresh (n = 65 101)
Race and ethnicity^a^			
African American or Black	1.68 (1.33-2.11)	1.78 (1.41-1.76)	1.06 (0.98-1.13)
Asian	0.93 (0.80-1.07)	0.84 (0.77-0.91)	0.79 (0.74-0.84)
Hispanic or Latino	1.47 (1.19-1.82)	1.65 (1.48-1.84)	1.55 (1.42-1.68)
Other or unknown^b^	1.25 (1.14-1.36)	0.97 (0.92-1.02)	0.83 (0.80-0.86)
Age, y^c^			
35-37	0.85 (0.77-0.95)	1.36 (1.28-1.45)	1.83 (1.76-1.91)
38-40	0.71 (0.64-0.79)	1.72 (1.59-1.86)	4.64 (4.36-4.92)
41-50	0.55 (0.48-0.63)	1.76 (1.60-1.95)	5.13 (4.69-5.61)
Prior birth events, No.^d^			
1	0.76 (0.68-0.84)	0.78 (0.73-0.83)	0.91 (0.87-0.95)
≥2	1.14 (0.97-1.34)	1.01 (0.92-1.12)	1.19 (1.10-1.29)
Oocytes retrieved, No.^e^			
6-10	1.64 (1.30-2.07)	1.61 (1.44-1.79)	1.98 (1.85-2.12)
11-15	2.14 (1.71-2.69)	1.93 (1.74-2.15)	1.90 (1.78-2.04)
≥16	3.26 (2.62-4.06)	2.03 (1.84-2.24)	1.53 (1.43-1.64)
Indication for ART^f^			
Male factor	0.75 (0.62-0.89)	1.05 (0.94-1.18)	0.97 (0.90-1.05)
Ovulation disorder	0.71(0.57-0.88)	1.22 (1.08-1.38)	1.01 (0.92-1.10)
Tubal factor	0.91 (0.71-1.17)	1.30 (1.14-1.48)	1.03 (0.94-1.12)
Uterine factor	0.73 (0.49-1.07)	1.32 (1.06-1.65)	0.73 (0.62-0.86)
Endometriosis	0.87 (0.64-1.19)	1.30 (1.11-1.52)	1.11 (1.00-1.23)
Unexplained	0.80 (0.68-0.94)	1.06 (0.94-1.18)	0.87 (0.81-0.94)
Multiple indications	1.01 (0.89-1.15)	1.20 (1.08-1.32)	1.08 (1.01-1.16)
Other^g^	0.79 (0.65-0.97)	1.01 (0.89-1.17)	0.92 (0.82-1.16)
No indication	0.64 (0.46-0.90)	1.25 (1.04-1.49)	0.80 (0.69-0.94)
State insurance mandate^h^	0.76 (0.68-0.85)	0.63 (0.59-0.66)	0.47 (0.45-0.49)

Abbreviations: ART, assisted reproductive technology; FET, frozen embryo transfer; MET, multiple embryo transfer; OR, odds ratio; PGT-A, preimplantation genetic testing for aneuploidy.

^a^ The reference group was White individuals.

^b^ The other or unknown category comprises individuals who were not asked to self-identify, who declined to self-identify, who self-identified as multiple races, or who identified as a racial group that constituted a small portion of the overall database.

^c^ The reference group was individuals aged 18 to 34 years.

^d^ The reference group was individuals with 0 prior birth events.

^e^ The reference group was individuals with 1 to 5 oocytes retrieved.

^f^ The reference group was individuals with diminished ovarian reserve.

^g^ Given the small number of cycles performed in which the ART indication was PGT and no infertility indication, these categories were grouped into other.

^h^ The reference group was individuals in states with no mandated insurance.

Across transfer types, having more oocytes retrieved was associated with increased odds of MET; for PGT-A cycles, the odds of MET increased with each increment in oocytes retrieved. For example, when 16 or more oocytes were retrieved, the odds of MET were more than 3 times higher compared with receiving 1 to 5 oocytes (OR, 3.26; 95% CI, 2.62-4.06). For all cycle types, mandated insurance was associated with decreased odds of MET. For PGT-A cycles, the odds of MET were 24% lower in states without an insurance mandate (OR, 0.76; 95% CI, 0.68-0.85).

Across cycle types, odds of live birth were higher after MET than SET. For example, for FET cycles with PGT-A, the odds of live birth were 70% higher with MET (OR, 1.70; 95% CI, 1.61-1.78, Table 3). African American individuals had lower odds of live birth compared with White individuals in all transfer types (FET with PGT-A: OR, 0.71; 95% CI, 0.52-0.90; untested FET: OR, 0.74; 95% CI, 0.63-0.85; untested fresh: OR, 0.71; 95% CI, 0.63-0.79). Individuals older than age 35 years had lower odds of live birth for untested cycles (eg, for ages 38-40 years, the OR was 0.50; 95% CI, 0.43-0.58 for untested FET and 0.46; 95% CI, 0.41-0.52 for untested fresh cycles) but not for cycles with PGT-A.

**Table 3.** Logistic Regression Models for Live Birth

Characteristic	Live birth, OR (95% CI)		
	FET with PGT-A (n = 13 695)	Untested FET (n = 21 091)	Untested fresh (n = 55 197)
MET^a^	1.70 (1.61-1.78)	1.55 (1.50-1.60)	1.37 (1.34-1.40)
Race^b^			
African American or Black	0.71 (0.52-0.90	0.74 (0.63-0.85)	0.71 (0.63-0.79)
Asian	1.04 (0.95-1.15)	0.96 (0.88-1.05)	0.68 (0.62-0.74)
Hispanic or Latino	0.95 (0.79-1.12)	0.85 (0.74-0.96)	0.90 (0.83-0.98)
Other or unknown^c^	1.15 (1.08-1.21)	1.02 (0.96-1.07)	0.95 (0.91-0.98)
BMI^d^	0.99 (0.98-0.99)	0.98 (0.97-0.98)	0.98 (0.98-0.98)
Age, y^e^			
35-37	1.05 (0.97-1.14)	0.74 (0.68-0.80)	0.75 (0.71-0.79)
38-40	1.03 (0.95-1.12)	0.50 (0.43-0.58)	0.46 (0.41-0.52)
41-50	0.91 (0.81-1.01)	0.27 (0.16-0.38)	0.18 (0.09-0.27)
Prior birth events, No.^f^			
1	1.01 (0.94-1.09)	1.04 (0.97-1.10)	1.14 (1.09-1.18)
≥2	0.91 (0.78-1.03)	1.04 (0.93-1.14)	1.07 (1.00-1.15)
Indication for ART^g^			
Male factor	1.13 (1.00-1.26)	1.28 (1.17-1.39)	1.28 (1.20-1.35)
Ovulation disorder	1.03 (0.87-1.19)	1.04 (0.92-1.16)	1.24 (1.16-1.32)
Tubal factor	0.98 (0.79-1.18)	1.08 (0.94-1.21)	1.13 (1.05-1.22)
Uterine factor	0.66 (0.38-0.94)	0.81 (0.58-1.04)	0.96 (0.80-1.12)
Endometriosis	0.85 (0.62-1.09)	1.07 (0.91-1.23)	1.28 (1.18-1.38)
Unexplained	1.09 (0.97-1.21)	1.11 (1.00-1.23)	1.16 (1.09-1.23)
Multiple indications	0.99 (0.89-1.08)	1.12 (1.01-1.22)	1.10 (1.03-1.17)
Other^h^	0.96 (0.82-1.10)	1.11 (0.97-1.26)	1.08 (0.97-1.19)
No indication	1.22 (0.98-1.46)	1.19 (1.01-1.36)	1.47 (1.32-1.62)
State insurance mandate^i^	1.07 (1.00-1.15)	1.13 (1.08-1.19)	1.04 (1.00-1.07)

Abbreviations: ART, assisted reproductive technology; BMI, body mass index (calculated as weight in kilograms divided by height in meters squared); FET, frozen embryo transfer; MET, multiple embryo transfer; OR, odds ratio; PGT-A, preimplantation genetic testing for aneuploidy.

^a^ The reference group was individuals with 1 embryo transferred.

^b^ The reference group was White individuals.

^c^ The other or unknown category comprises individuals who were not asked to self-identify, who declined to self-identify, who self-identified as multiple races, or who identified as a racial group that constituted a small portion of the overall database (American Indian or Alaskan Native and Native Hawaiian or other Pacific Islander).

^d^ Per 1-unit increase in BMI.

^e^ The reference group was individuals aged 18 to 34 years.

^f^ The reference group was individuals with 0 prior birth events.

^g^ The reference group was individuals with diminished ovarian reserve.

^h^ Given the small number of cycles performed in which the ART indication was PGT and no infertility indication, these categories were grouped into other.

^i^ The reference group was individuals in states with no mandated insurance.

Among 53 880 cycles with a live birth, 996 of 9712 FET cycles with PGT-A (10.3%) had multiples at birth, half the rates for untested fresh (2816 of 13765 cycles [20.5%]) and frozen (6714 of 30403 cycles [22.1%]) embryo transfer cycles. Among 27 173 cycles with a live birth from an SET, less than 2% of cycles had multiples (436 cycles [1.6%]).

The variable associated with the greatest increase in odds of having multiple liveborn infants at delivery was MET; for example, the odds of having multiples were more than 5 times higher (OR, 5.33; 95% CI, 5.22-5.44) for individuals who underwent PGT-A with MET compared with those who underwent SET (Table 4). For FET with PGT-A, there was no difference in odds of multiple gestation delivery by age category. However, for untested fresh and frozen embryo transfers, the risk of multiple gestation delivery decreased with age in a stepwise manner. Compared with individuals aged 18 to 34 years, those aged 41 to 50 years undergoing untested FET had 25% lower odds of multiple gestation delivery (OR, 0.75; 95% CI, 0.59-0.91) and those undergoing untested fresh embryo transfer had 35% lower odds of multiple gestation delivery (OR, 0.65; 95% CI, 0.53-0.78).

**Table 4.** Logistic Regression Models for Multiple Gestation at Birth

Characteristic	Multiple gestation delivery, OR (95% CI)		
	FET with PGT-A (n = 7551)	Untested FET (n = 10 458)	Untested fresh (n = 25 763)
MET^a^	5.32 (5.21-5.44)	3.24 (3.15-3.33)	3.26 (3.20-3.32)
Race^b^			
African American or Black	0.90 (0.51-1.29)	0.97 (0.81-1.13)	0.95 (.083-1.06)
Asian	1.04 (0.87-1.20)	0.91 (0.76-1.05)	0.90 (0.80-1.01)
Hispanic or Latino	1.03 (0.75-1.30)	0.87 (0.69-1.05)	0.98 (0.87-1.10)
Other or unknown^c^	1.05 (0.92-1.17)	0.93 (0.83-1.03)	1.01 (0.96-1.06)
BMI^d^	1.01 (1.00-1.03)	1.01 (1.00-1.01)	1.01 (1.00-1.01)
Age, y^e^			
35-37	0.97 (0.83-1.11)	0.88 (0.78-0.98)	0.88 (0.81-0.94)
38-40	1.01 (0.87-1.15)	0.81 (0.70-0.93)	0.69 (0.61-0.77)
41-50	1.01 (0.82-1.21)	0.75 (0.59-0.91)	0.65 (0.53-0.78)
Prior birth events, No.^f^			
1	1.01 (0.85-1.18)	1.04 (0.94-1.14)	1.01 (0.94-1.08)
≥2	0.97 (0.69-1.25)	1.01 (0.84-1.18)	1.08 (0.97-1.19)
Indication for ART^g^			
Male factor	1.05 (0.83-1.27)	1.09 (0.89-1.28)	1.12 (1.00-1.24)
Ovulation disorder	1.00 (0.69-1.30)	1.11 (0.92-1.30)	1.09 (0.9-1.22)
Tubal factor	1.09 (0.74-1.44)	1.02 (0.78-1.25)	1.13 (0.96-1.29)
Uterine factor	1.12 (0.6501.59)	0.88 (0.55-1.22)	1.06 (0.80-1.32)
Endometriosis	1.00 (0.60-1.41)	1.11 (0.83-1.40)	1.09 (0.94-1.23)
Unexplained	0.99 (0.78-1.21)	1.04 (0.80-1.28)	1.11 (0.99-1.23)
Multiple indications	1.00 (0.85-1.18)	1.09 (0.90-1.28)	1.11 (1.00-1.22)
Other^h^	0.99 (0.72-1.25)	1.00 (0.77-1.24)	1.13 (0.97-1.29)
No indication	1.17 (0.74-1.61)	1.07 (0.75-1.38)	1.08 (0.83-1.33)
State insurance mandate^i^	0.98 (0.83-1.13)	1.01 (0.92-1.10)	0.98 (0.92-1.04)

Abbreviations: ART, assisted reproductive technology; BMI, body mass index (calculated as weight in kilograms divided by height in meters squared); FET, frozen embryo transfer; MET, multiple embryo transfer; OR, odds ratio; PGT-A, preimplantation genetic testing for aneuploidy.

^a^ The reference group was individuals with 1 embryo transferred.

^b^ The reference group was White individuals.

^c^ The other or unknown category comprises individuals who were not asked to self-identify, who declined to self-identify, who self-identified as multiple races, or who identified as a racial group that constituted a small portion of the overall database (American Indian or Alaskan Native and Native Hawaiian or other Pacific Islander).

^d^ Per 1-unit increase in BMI.

^e^ The reference group was individuals aged 18 to 34 years.

^f^ The reference group was individuals with 0 prior birth events.

^g^ The reference group was individuals with diminished ovarian reserve.

^h^ Given the small number of cycles performed in which the ART indication was PGT and no infertility indication, these categories were grouped into other.

^i^ The reference group was individuals in states with no mandated insurance.

## Discussion

This cohort study found that while cycles using PGT-A were less likely than untested cycles to use MET or have a multiple gestation pregnancy, MET was still a common practice in PGT-A cycles in the US. Insurance mandates were associated with reduced risk of MET in PGT-A cycles. We also found that compared with White individuals, African American and Hispanic individuals were more likely to undergo MET regardless of PGT-A use.

Responding to the risk of multiple gestations associated with MET, ASRM released its first embryo transfer guidelines in 1998.^8^ The introduction of these guidelines coincided with a decrease in higher-order multiple gestations (ie, triplets or higher) resulting from IVF, but there was little change in the incidence of twin births after IVF.^1,11,12^ Since 1998, updated guidelines have placed increased emphasis on SET, and the most recent guidelines recommend that only 1 euploid embryo be transferred at a time.^9^

Clinicians are more likely to deviate from ASRM or SART guidelines at patient request or when working with patients with previously failed IVF cycles.^24^ Factors associated with patient preference for MET include lack of IVF insurance coverage,^16,25^ limited resources to pursue multiple IVF cycles,^26^ and a desire for multiple gestation.^27^ Some patients want to complete family building more expeditiously, which may be associated with a desire for multiple gestation,^27^ and this may be more pronounced at older ages.^28^

Despite improvements in the success of IVF treatments over the years, the odds of a live birth after 1 oocyte retrieval for IVF remain low and the risk of multiple gestations is high.^29,30^ The promise of PGT-A was to improve the efficiency and safety of IVF by selecting euploid embryos for SET. To the contrary, we found that MET was not uncommon in PGT-A cycles. Furthermore, a recent randomized trial by Yan et al^31^ found no improvement in cumulative live birth rates after IVF with PGT-A. Unlike the Yan et al study, most prior work that found improvements associated with PGT-A limited analysis to live birth rate per embryo transfer rather than cumulative live birth rate per initiated treatment cycle. Results from Yan et al^31^ highlight that PGT-A can be invasive and may yield false-positive results leading to embryo wastage. Altogether, these findings raise the question of why PGT-A use has increased dramatically in recent years.^31^

Over the last several years, an increasing number of states have required that insurance cover IVF.^21^ Despite this development, the cost of fertility treatment is largely self-funded. The mean cost of IVF is approximately $19 000,^32^ and therefore cost, especially out-of-pocket cost, may be associated with medical practice and outcomes. Our group previously found that IVF insurance was independently associated with an increased cumulative chance of live birth for individuals who required IVF to conceive simply because these individuals were more likely to pursue additional IVF cycles after a failed cycle.^33,34^ On a national level, fertility practices and patient decisions regarding fertility care vary by presence or absence of state-mandated insurance. In a 2002 study, Jain et al^16^ found that more embryos were transferred per cycle in states without insurance mandates. More recently, Styer et al^25^ found that elective SET was more likely to be performed in states with a mandate. Similarly, we found that MET was more common after PGT-A in states without insurance mandates. In a recent study^19^ of SART data, our group also found that the proportion of IVF cycles using PGT-A was higher in states without IVF insurance mandates than in states with mandates. While this adds to patient cost for IVF,^35^ patients and clinicians in nonmandate states may be under increased pressure to maximize live birth rate per transfer.

Our findings suggest that individuals older than age 35 years were less likely to undergo MET during PGT-A than those ages 18 to 34 years, and in untested fresh and frozen cycles, they were more likely to undergo MET. This may reflect a decreasing number of euploid embryos with age; for PGT-A cycles, there may be fewer confirmed euploid embryos to transfer, and for untested cycles, more embryos may be transferred given overall worse prognosis. We also found that African American and Hispanic individuals were more likely to undergo MET than White individuals. A previous study^25^ found that African American and Hispanic individuals were less likely to choose SET. Use of more than 1 embryo may be associated with worse outcomes after IVF among African American and Hispanic compared with White individuals, including lower rates of pregnancy and higher rates of pregnancy loss.^36,37^ Indeed, African American and Hispanic individuals have been found to have lower rates of pregnancy after SET.^38^ Previous work by our group^39^ found that African American individuals were less likely to pursue additional cycles of IVF than White individuals if their first cycle was unsuccessful. African American individuals may choose MET to achieve higher pregnancy rates knowing that they are unlikely to continue treatment. Given the large percentage of cycles with missing race and ethnicity data in our study and others, these interpretations should be made cautiously.^40^ Nevertheless, these studies highlight significant disparities in access to fertility care and fertility practices that must be addressed to improve outcomes in this population.

MET was the factor associated with greatest increase in odds of multiple gestation. For untested cycles, older individuals had a lower chance of multiples than those younger than age 34 years even after controlling for MET. However, this trend was not found for PGT-A cycles. Furthermore, among individuals undergoing SET, use of PGT-A was associated with decreased odds of twins. Other studies have found an increased risk of monozygotic twinning after SET with increasing patient age and poor embryo quality.^41,42^ Our study is the first study, to our knowledge, to find an association between PGT-A and monozygotic twinning. Possible explanations may include laboratory practices avoiding biopsy and cryopreservation of embryos with poor quality and antepartum elective or spontaneous reduction of multiple gestations.

This study has several strengths. SART collects data on IVF cycles from more than 90% of IVF centers in the US, making this data set a representative sample of IVF cycles performed in the US between 2014 and 2016. The large sample size and population-based study design may give our study further generalizability.

### Limitations

Several limitations of this study must be considered. First, it is unknown whether the transfer of 1 embryo was elective (ie, whether a choice was made to have 1 embryo transferred or if there was only 1 embryo available). Second, it is unknown whether singleton gestations at birth originated as multiples and underwent selective or spontaneous reduction prior to 20 weeks, and the zygosity of multiples was also unknown. Third, SART does not collect patient-level insurance information, and thus we based IVF insurance status on the state mandate under which the cycle was performed. However, patients living in nonmandated states may have had IVF insurance coverage through their employers or may have traveled across state lines for care. However, state mandates were likely associated with clinician practice. Fourth, a large portion of cycles had missing race and BMI data. This is a known limitation of SARTdata.^40^ Fifth, the data set was limited to 2014 to 2016 given that SART does not release updated data to researchers without a new application; therefore, it is not clear if these practice patterns persist. However, this research was novel, and to our knowledge, no other investigations have brought to light the concern for multiple embryo transfer after PGT-A. It is not clear if clinicians treating individuals without IVF insurance coverage would move to change practice patterns without recognizing that the problem exists.

## Conclusions

While this cohort study found that PGT-A was associated with higher rates of use of SET, a substantial portion of transfers in the US were of multiple embryos even after PGT-A. Although MET was associated with higher odds of live birth, it was also associated with significantly higher odds of multiple live births. Cycles conducted in states without insurance mandates were more likely to use riskier embryo-transfer practices. Altogether, our findings suggest the need for effective and up-to-date IVF insurance policy across the country to help maximize evidence-based decision-making for people with infertility given that legislative efforts to increase state-mandated insurance may be associated with improved delivery and outcomes of IVF.

## Notes

Supplement 1. **eFigure.** Flow Chart of Included Cycles in Data Set **eTable.** Mean Number of Embryos Transferred by Age and Cycle Type Categories

Supplement 2.
Data Sharing Statement
