# Supplementary material for: Association of State Insurance Mandates for Fertility Treatment With Multiple Embryo Transfer After Preimplantation Genetic Testing for Aneuploidy
Source: JAMA Netw Open. 2023 Jan 27;6(1):e2251739. doi: 10.1001/jamanetworkopen.2022.51739 (PMC12549158; doi:10.1001/jamanetworkopen.2022.51739)
Supplement: Supplement 1. — eFigure. Flow Chart of Included Cycles in Data Set eTable. Mean Number of Embryos Transferred by Age and Cycle Type Categories [file jamanetwopen-e2251739-s001.pdf]

Supplemental Online Content

Bedrick BS, Nickel KB, Riley JK, Jain T, Jungheim ES. Association of state insurance mandates for fertility treatment with multiple embryo transfer after preimplantation genetic testing for aneuploidy. *JAMA Netw Open*. 2023;6(1):e2251739.
doi:10.1001/jamanetworkopen.2022.51739

eFigure. Flow Chart of Included Cycles in Data Set
eTable. Mean Number of Embryos Transferred by Age and Cycle Type Categories

This supplemental material has been provided by the authors to give readers additional information about their work.

© 2023 Bedrick BS et al. *JAMA Network Open.*

eFigure. Flow Chart of Included Cycles in Data Set

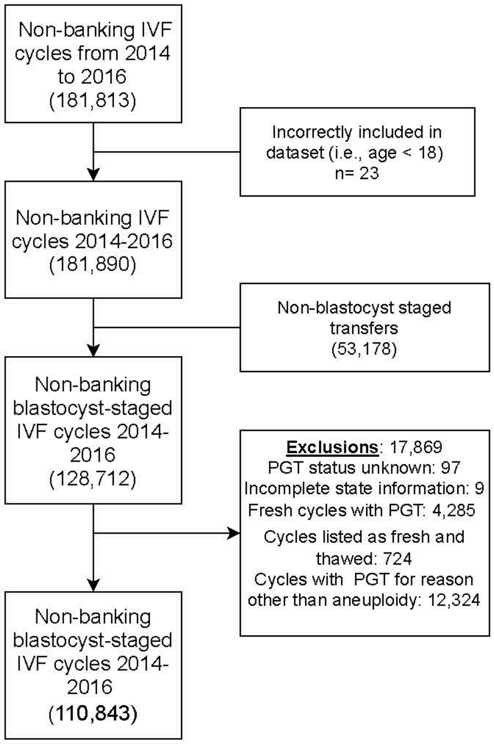

© 2023 Bedrick BS et al. *JAMA Network Open.*

eTable. Mean Number of Embryos Transferred by Age and Cycle Type Categories

Cycle Type	PGT-A FET	Untested FET	Untested Fresh
Total N	17,650	28,046	65,147
Mean number of embryos transferred, (SD)	1.2 (0.4)	1.5 (0.6)	1.6 (0.6)
18-34 years	1.2 (0.4)	1.5 (0.5)	1.5 (0.5)
35-37 years	1.2 (0.4)	1.5 (0.5)	1.6 (0.5)
38-40 years	1.2 (0.4)	1.6 (0.6)	1.9 (0.6)
41-50 years	1.1 (0.4)	1.7 (0.7)	2.2 (0.9)

Abbreviations: FET, frozen embryo transfer; PGT, Preimplantation Genetic Testing; PGT-A, Preimplantation Genetic Testing for Aneuploidy; SD, standard deviation

© 2023 Bedrick BS et al. *JAMA Network Open.*
