# Supplementary material for: Association of State Insurance Mandates for Fertility Treatment With Multiple Embryo Transfer After Preimplantation Genetic Testing for Aneuploidy
Source: JAMA Netw Open. 2023 Jan 27;6(1):e2251739. doi: 10.1001/jamanetworkopen.2022.51739 (PMC12549158; doi:10.1001/jamanetworkopen.2022.51739)
Supplement: Supplement 2. — Data Sharing Statement [file jamanetwopen-e2251739-s002.pdf]

Data Sharing Statement

Bedrick BS, Nickel KB, Riley JK, Jain T, Jungheim ES. Association of state insurance mandates for fertility treatment with multiple embryo transfer after preimplantation genetic testing for aneuploidy. *JAMA Netw Open*. 2023;6(1):e2251739. doi:10.1001/jamanetworkopen.2022.51739

Data
Data available: No

Additional Information
Explanation for why data not available: The data is available via application to SART. The authors do not have the authority to share it.

© 2023 Bedrick BS et al. *JAMA Network Open*.
